# Designing thin film materials — Ternary borides from first principles

**DOI:** 10.1016/j.tsf.2015.03.035

**Published:** 2015-05-29

**Authors:** H. Euchner, P.H. Mayrhofer

**Affiliations:** Institute of Materials Science and Technology, Vienna University of Technology, 1040 Vienna, Austria

**Keywords:** Ternary systems, Diborides, Phase stability, Ab initio simulation

## Abstract

Exploiting the mechanisms responsible for the exceptional properties of aluminum based nitride coatings, we apply ab initio calculations to develop a recipe for designing functional thin film materials based on ternary diborides. The combination of binary diborides, preferring different structure types, results in supersaturated metastable ternary systems with potential for phase transformation induced effects. For the exemplary cases of M*_x_*W_1 − *x*_B_2_ (with M = Al, Ti, V) we show by detailed ab initio calculations that the respective ternary solid solutions are likely to be experimentally accessible by modern depositions techniques.

## Highlights

• Interplay and competition of two different structural modifications in ternary borides
• Stability of ternary boride systems with respect to spinodal decomposition
• Enormous potential of ternary borides for thin film applications

## 1 Introduction

The increasing demand in industrial applications calls for new protective coatings with high hardness, good elastic properties and improved ductility. Transition metal (TM) nitrides have proven to fulfill these requirements for different kinds of applications like automotive or aerospace industries. Nevertheless, the search for improved materials is an ongoing topic being far from its end.

It is well studied that the face centered Ti_1 − *x*_Al*_x_*N (B1 structure, rocksalt NaCl prototype), being the basis of many hard coatings [1], is a supersaturated structure and tends to decompose into the stable constituents, cubic TiN (B1) and hexagonal AlN (B4 structure, wurtzite ZnS prototype) [2,3]. The ability of Ti_1 − *x*_Al*_x_*N to crystallize in a single phase cubic structure, when synthesized by vapor phase deposition techniques [4] allows combining several advantages. The cubic phase has superior physical and mechanical properties like hardness and elastic moduli as compared to the wurtzite structure. Additionally, with increasing Al content, the oxidation resistance is strongly improved [1]. Moreover, with higher Al content, the supersaturation of the TiN-based cubic structure increases, which allows for self-hardening effect at elevated temperatures, resulting from the formation of TiN- and AlN-rich cubic domains [5,2,3]. Finally the transition from cubic to wurtzite AlN, which takes place upon further annealing or exposure to elevated temperatures, which typically results in a loss of mechanical properties, may yield a phase transformation induced toughening effect for controlled AlN phase fractions [6]. The latter is based on the enormous 26% volume increase upon transformation from the metastable cubic to the stable hexagonal structure. Consequently, it can be concluded that the unique properties of Ti_1 − *x*_Al*_x_*N originate from the interplay and competition between two phases that prefer different structure types — cubic TiN and wurtzite AlN.

Boron on the other hand is one of the hardest materials known. Therefore, a promising pathway to achieve strong materials with exceptional properties is the investigation of borides, which have attracted increasing interest in recent years. However, while ternary and even quaternary transition metal (TM) nitrides have been investigated in detail, borides and especially multinary borides are still rather unexplored. Consequently, we present first theoretical predictions for the development of single-phased ternary TM-diborides. These predictions are based on the well-studied mechanisms which are responsible for the exceptional properties of supersaturated cubic-structured Ti_1 − *x*_Al*_x_*N.

**Fig. 1:**
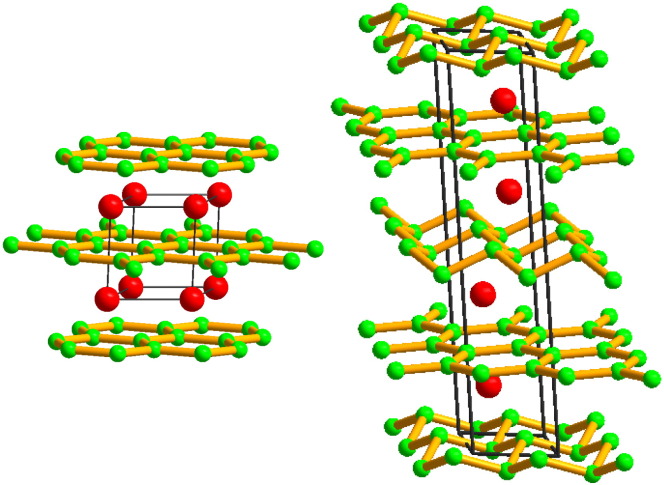
Layer structure of AlB_2_ (left) and WB_2_ (right) prototypes.

A large number of diborides, including the early transition metal (TM) diborides, crystallize in the so-called AlB_2_ structure type [7] with space group 191 (*P*6/*mmm*) and three atoms per hexagonal unit cell. In addition to the unit cell description it is instructive to represent the structure as a stacking of hexagonal planes of covalently bonded boron atoms, separated by the metal layers, as depicted in Fig. 1. While the boron layers consist of graphite-like hexagons, the metal atoms are located above (and below) the centers of these hexagons. Despite the fact that the AlB_2_ structure type is the predominant one, there also exist diboride phases which prefer to crystallize in other modifications. One such phase is WB_2_, for which two structural modifications are reported [8–10]. While, recently thin films of WB_2_ have been reported to crystallize in the AlB_2_ structure type, bulk material seems to prefer the WB_2_ structure type, formerly known as W_2_B_5_. The WB_2_ structure type is closely related to the AlB_2_ prototype but evidences a different layer structure. In fact, WB_2_ consists of both flat and puckered boron layers, resulting in a twelve atom unit cell with space group 194 (*P*63/*mmc*) as depicted in Fig. 1.

Due to the existence of the different structural modifications, combining AlB_2_-structured TM-diborides with WB_2_ will lead to ternary model systems that are based on competing allotropes. In the following, three examples of such ternary model systems will be discussed with respect to formation and stability. As mentioned above, for WB_2_ both, the AlB_2_ (WB_2_-191) and WB_2_ prototype (WB_2_-194) are reported. [8–10]. To distinguish these structural modifications, we use *a* and *w* to represent the AlB_2_ (*a*-M*_x_*W_1−*x*_B_2_) and the WB_2_ prototype (*w*-M*_x_*W_1−*x*_B_2_), respectively.

## 2 Computational methods

To investigate the respective stability of different metal diborides, MB_2_ (with M = Al, Ti, V) and WB_2_ in the *a*- and *w*-modifications, density functional theory (DFT) calculations have been conducted. The Vienna Ab Initio Simulation Package (VASP) [11–13] was used to optimize the respective structures, applying the projector augmented wave method within the generalized gradient approximation (PAW-GGA). The calculated total energies of the respective allotropes are denoted in Table 1. As expected *a*-MB_2_ exhibits a more negative total energy than *w*-MB_2_, whereas for WB_2_ the opposite is true, meaning the total energy of *w*-WB_2_ is more negative. Thus, as previously discussed in literature [14] we also find WB_2_ to be energetically more stable in the WB_2_ structure type, while MB_2_ is stable in the AlB_2_ structure type.

The absolute values of the energy differences between the *a*- and *w*-allotropes of AlB_2_, TiB_2_, VB_2_, and WB_2_ are of about 113, 390, 94, and 260 meV/at, respectively, which is in the same range as for the wurtzite and cubic modification of AlN. Moreover, a volume increase of about 6.5–9.5% is evidenced for the allotropic transformation of *a*-MB_2_ to *w*-MB_2_, which may be relevant for increasing fracture toughness. As a consequence M*_x_*W_1 − *x*_B_2_ alloys are promising candidates for more detailed studies.

For an investigation of ternary M*_x_*W_1 − *x*_B_2_ phases, supercell structures of both structural modifications were constructed. In the case of *a*-M*_x_*W_1 − *x*_B_2_ a 4 × 4 × 2 supercell with 96 atoms was investigated, while for *w*-M*_x_*W_1 − *x*_B_2_ a 4 × 2 × 1 supercell, again containing 96 atoms, was selected. The respective metal sublattices were then populated by different M/W contents, making use of the special quasirandom structure (SQS) approach [15,16]. The obtained SQS structures were then optimized by means of DFT, applying an energy cutoff of 600 eV and a 4 × 4 × 8 Γ-centered k-point mesh for *a*-M*_x_*W_1 − *x*_B_2_, whereas in the case of *w*-M*_x_*W_1 − *x*_B_2_ a corresponding 8 × 4 × 4 k-point mesh was used. Energy cutoff and k-point mesh were carefully chosen to ensure energy convergence within an accuracy of about 1 meV/at.

## 3 Results and discussion

To determine the respective stability of both structural modifications of M*_x_*W_1 − *x*_B_2_ at a given metal concentration, the energy of formation, *E_f_*, was calculated following Eq. (1):

(1) $$E_{f}=\frac{1}{\sum_{i}n_{i}}\;\left(E_{\mathrm{tot}}-\sum_{i}n_{i}E_{i}\right)$$

with *E_tot_* and *E_i_* the total energy of the compound and its elemental constituents, as determined from DFT and *n_i_* the number of atoms of species *i*. Thus, in our case, the energy of formation describes the energy that is gained when an M*_x_*W_1 − *x*_B_2_ alloy is formed from *α*-boron, bcc-W and the corresponding metal (fcc-Al, bcc-V and *α*-Ti).

**Fig. 2:**
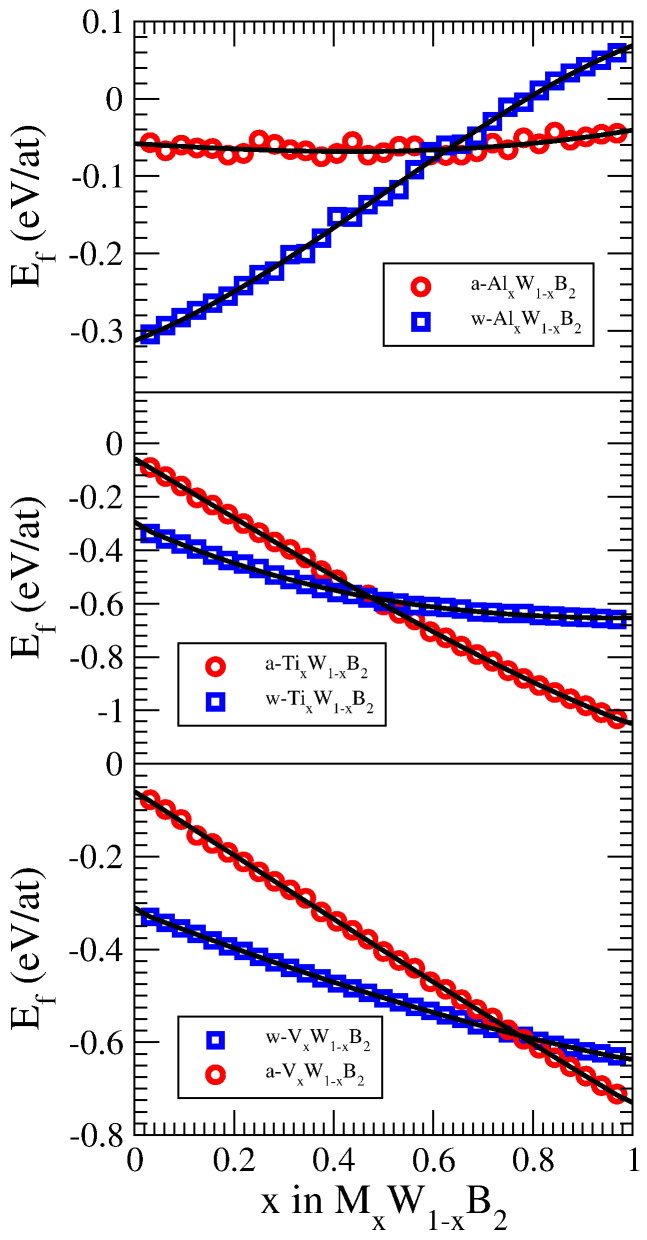
Energy of formation of M*_x_*W_1 − *x*_B_2_ in the respective allotropic modification (red circles: *a*-M*_x_*W_1 − *x*_B_2_, blue squares: *w*-M*_x_*W_1 − *x*_B_2_). The black curves are fits to the data.

The energy of formation of Al*_x_*W_1 − *x*_B_2_ is depicted in Fig. 2 (top panel). With up to an Al content of about 60% on the metal sublattice we clearly find *w*-Al*_x_*W_1 − *x*_B_2_ to be favored, while at higher Al content *a*-Al*_x_*W_1 − *x*_B_2_ becomes more stable. Interestingly, the energy of formation of *a*-Al*_x_*W_1 − *x*_B_2_ is almost constant over the whole composition range, while *w*-Al*_x_*W_1 − *x*_B_2_ even becomes unstable against decomposition into fcc-aluminum and *α*-boron at about 70% Al. In the case of Ti*_x_*W_1 − *x*_B_2_ the cross-over from *w*- to *a*-phase is located at a Ti content of about 45%, while for V*_x_*W_1 − *x*_B_2_ the *a*-phase gets stabilized at about 75% V content (see Fig. 2).

To ensure the mechanical stability of the investigated structures the stability criterion for hexagonal crystals must be fulfilled [17]:

(2) $$C_{11}\geq C_{12}\;C_{44}\geq 0\;C_{11}C_{33}\geq C_{13}^{2}\text{.}$$

Therefore, the elastic constants of several representative configurations were determined. For this purpose the stress–strain relation was used, together with the universal linear independent coupling strain approach as introduced by Yu et al. [18]. Following this approach, six linear independent strains were applied to the respective cell, thus resulting in six different strain states for each investigated configuration. After relaxation of the atomic positions of these strain states at fixed lattice vectors, the corresponding stresses were determined. The optimization of the strained configurations was again conducted using the VASP code, applying the same settings as described in Section 2. Then the stress–strain relation was evaluated and the elastic constants were obtained by linear least square fits using single value decomposition [18]. In a last step the hexagonal analog of the elastic tensor was determined [19]. This is necessary since the underlying lattice of the random alloys may slightly deviate from hexagonal symmetry. The resulting elastic constants for all binaries and the intermediate ternaries are denoted in Tables 2 and 3. As can be easily verified, the elastic constants of the investigated configurations indeed fulfill the stability criterion for both structure types. The fact that even the less stable binaries fulfill the stability criterion strongly indicates that the latter one will not be violated for any other configuration.

**Fig. 3:**
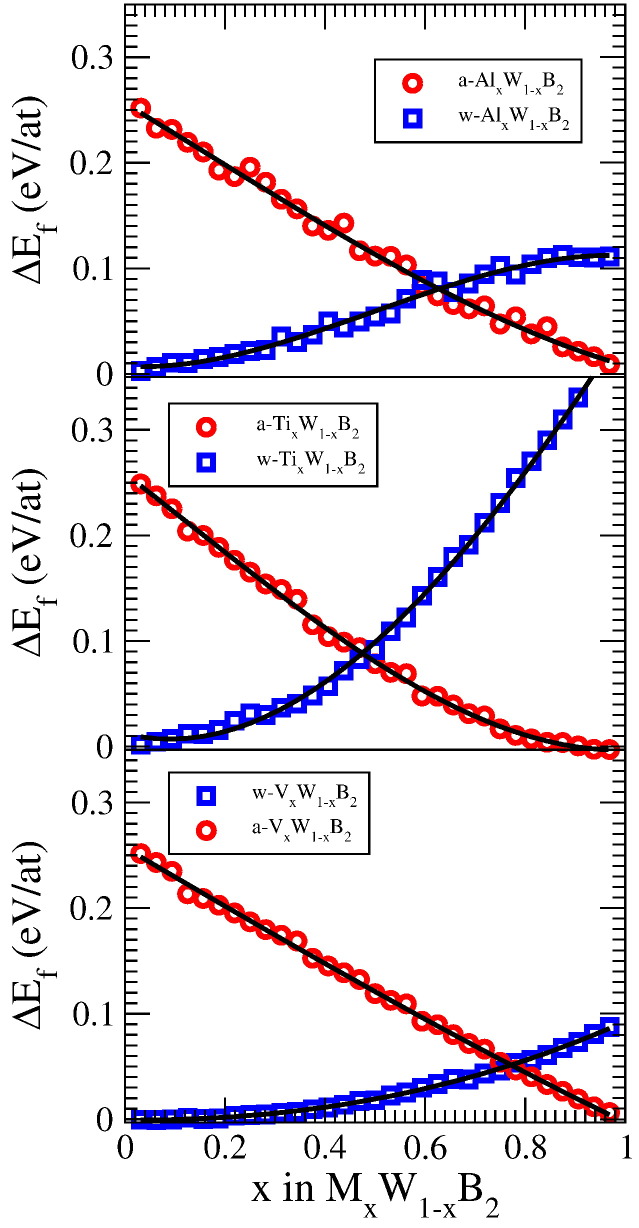
Difference in energy of formation of M*_x_*W_1 − *x*_B_2_ with respect to the stable constituents *a*-MB_2_ and *w*-WB_2_ (red circles: *a*-M*_x_*W_1 − *x*_B_2_, blue squares: *w*-M*_x_*W_1 − *x*_B_2_). The black curves are fits to the data.

The mixing enthalpy of the supersaturated *a*-M*_x_*W_1 − *x*_B_2_ and *w*-M*_x_*W_1 − *x*_B_2_ phases – with respect to the energetically stable constituents *a*-MB_2_ and *w*-WB_2_ – takes positive values for the whole composition range, as is depicted in Fig. 3. Hence, a broad miscibility gap is present, yet, the energy differences are small and comparable to those in the Ti_1 − *x*_Al*_x_*N system [20–22]. As a consequence, the supersaturated *a*-M*_x_*W_1 − *x*_B_2_ and *w*-M*_x_*W_1 − *x*_B_2_ solid solutions are likely to be accessible by experimental non-equilibrium growth techniques. Interestingly, recent experimental studies have even shown that WB_2_ thin films can be synthesized in the metastable AlB_2_ structure by physical vapor deposition (PVD) [10]. The decreasing energy difference between supersaturated *w*- and *a*-M*_x_*W_1 − *x*_B_2_ (see Fig. 3) clearly indicates that additional elements such as Al, Ti, or V will even further promote the crystallization of AlB_2_ structured *a*-M*_x_*W_1 − *x*_B_2_. An experimental report by Sobol et al. [23], which indeed proves that solid solutions of *a*-Ti*_x_*W_1 − *x*_B_2_ can be prepared by PVD, further corroborates this statement.

The above discussion is restricted to the enthalpy of mixing, since the exact thermodynamics of non-equilibrium processes such as PVD is not accessible. However, it has to be emphasized that the cross-over from *w*-M*_x_*W_1 − *x*_B_2_ to *a*-M*_x_*W_1 − *x*_B_2_ is not affected by entropic contributions. This is due to the configurational entropy of an ideal random solid solution depending only on the stoichiometry, such that at a given chemical composition it is equivalent for both structure types. Vibrational entropy on the other hand may slightly differ for the two structural modifications, but this difference may only be significant at high temperatures.

**Fig. 4:**
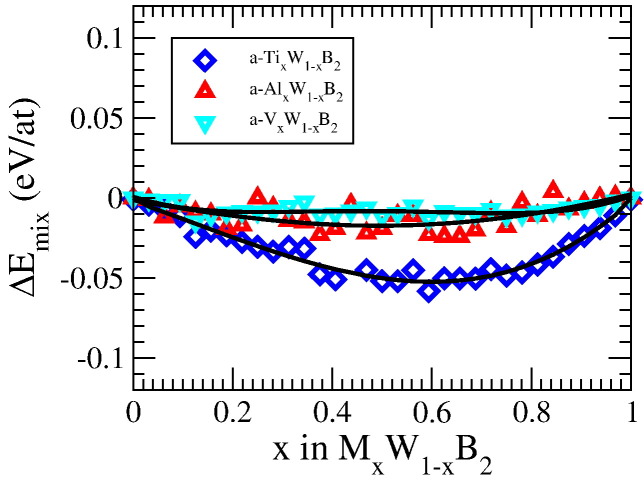
Enthalpy of mixing of M*_x_*W_1 − *x*_B_2_ with respect to the *a*-phase. The black curves are fits to the data.

Moreover, supersaturated solid solutions of *a*-M*_x_*W_1 − *x*_B_2_ may prove to be highly stable, since they essentially show no tendencies for spinodal decomposition. When plotting the differences in energy of formation between *a*-M*_x_*W_1 − *x*_B_2_ and the isostructural constituent phases *a*-AlB_2_ and *a*-WB_2_, it becomes evident that the *a*-phase is stable against isostructural decomposition since Δ*E_mix_* is negative (or very close to zero) for the whole composition range (see Fig. 4). This will be even further enhanced when configurational entropy is taken into account.

## 4 Conclusion

The fact that an interplay between two different allotropes may result in materials with improved physical properties is well known from the famous Ti_1 − *x*_Al*_x_*N system. There metastable solid solutions with up to 70% Al on the metal sublattice can be prepared in the cubic crystal system, showing improved hardness and better ductility. These improvements in the physical properties originate in the interplay between the preference for hexagonal and cubic structure type of AlN and TiN. Following this idea of competing structure types we have studied supersaturated solid solution of ternary diborides, which are based on binary constituents that in principle prefer to crystallize in different modifications. On the exemplary cases of Al*_x_*W_1 − *x*_B_2_, Ti*_x_*W_1 − *x*_B_2_, and V*_x_*W_1 − *x*_B_2_, we have shown that such ternary diborides represent a new class of metastable materials which offer a large field for further investigation. The recent successful deposition of *a*-WB_2_ together with the calculated formation energies allows us to conclude that solid solutions of *a*-M*_x_*W_1 − *x*_B_2_ type are experimentally accessible over a large composition range. Apart from studies of phase stability with respect to temperature and decomposition, it is of high interest to exploit this structural interplay for the whole series of diborides crystallizing in the AlB_2_ structure type (e.g. Ti, Zr, Hf, V, Nb). Moreover alloying of other allotropes such as ReB_2_ or OsB_2_ with diborides in the AlB_2_ structure would be possible, and due to the larger volume differences even desirable. However, Re and Os based diborides would become much more expensive.

## Figures and Tables

**Table 1:** Total energies for MB_2_ and WB_2_ in the respective allotropic modification.

Structure type	AlB_2_		WB_2_	
	E*_tot_* (eV/at)	Vol (Å^3^/at)	E*_tot_* (eV/at)	Vol (Å^3^/at)
AlB_2_	− 5.736	8.60	− 5.623	9.15
TiB_2_	− 8.102	8.58	− 7.712	9.38
VB_2_	− 8.161	7.87	− 8.067	8.39
WB_2_	− 8.777	8.94	− 9.037	9.30

**Table 2:** Elastic constants for selected stoichiometries of (Al,Ti,V)*_x_*W_1 − *x*_B_2_ in the AlB_2_ structure type.

Structure	C_11_ (in GPa)	C_12_ (in GPa)	C_13_ (in GPa)	C_33_ (in GPa)	C_44_ (in GPa)
*a*-AlB_2_	526.4	101.6	14.4	341.7	24.9
*a*-TiB_2_	634.4	62.1	100.2	447.1	252.6
*a*-VB_2_	663.5	111.6	120.5	476.1	218.1
*a*-WB_2_	602.5	141.5	231.0	409.8	123.5
*a*-Al_0.5_W_0.5_B_2_	493.7	151.9	127.7	322.7	78.8
*a*-Ti_0.5_W_0.5_B_2_	602.2	119.01	173.8	430.4	200.6
*a*-V_0.5_W_0.5_B_2_	601.5	138.9	194.0	421.8	160.7

**Table 3:** Elastic constants for selected stoichiometries of (Al,Ti,V)*_x_*W_1 − *x*_B_2_ in the WB_2_ structure type.

Structure	C_11_ (in GPa)	C_12_ (in GPa)	C_13_ (in GPa)	C_33_ (in GPa)	C_44_ (in GPa)
*w*-AlB_2_	436.1	70.5	22.9	374.4	31.8
*w*-TiB_2_	441.3	106.3	85.6	499.0	141.6
*w*-VB_2_	546.8	95.3	110.1	577.1	234.3
*w*-WB_2_	571.9	136.4	186.7	654.9	218.7
*w*-Al_0.5_W_0.5_B_2_	462.7	108.4	127.3	418.2	113.4
*w*-Ti_0.5_W_0.5_B_2_	542.0	100.0	133.1	586.5	234.8
*w*-V_0.5_W_0.5_B_2_	562.4	118.2	143.8	633.7	244.3
